# *Ehrlichia* effector TRP120 manipulates bacteremia to facilitate tick acquisition

**DOI:** 10.1128/mbio.00476-24

**Published:** 2024-03-19

**Authors:** Tsian Zhang, Rory C. Chien, Khemraj Budachetri, Mingqun Lin, Prosper Boyaka, Weiyan Huang, Yasuko Rikihisa

**Affiliations:** 1 Department of Veterinary Biosciences, The Ohio State University, Columbus, Ohio, USA; The University of Edinburgh, Edinburgh, United Kingdom

**Keywords:** *Ehrlichia japonica*, TRP120, bacteremia, tick acquisition/transmission, blood, mouse, monocytes

## Abstract

*Ehrlichia* species are obligatory intracellular bacteria that cause a potentially fatal disease, human ehrlichiosis. The biomolecular mechanisms of tick acquisition of *Ehrlichia* and transmission between ticks and mammals are poorly understood. *Ehrlichia japonica* infection of mice recapitulates the full spectrum of human ehrlichiosis. We compared the pathogenicity and host acquisition of wild-type *E. japonica* with an isogenic transposon mutant of *E. japonica* that lacks tandem repeat protein 120 (TRP120) (ΔTRP120). Both wild-type and ΔTRP120 *E. japonica* proliferated similarly in cultures of mammalian and tick cells. Upon inoculation into mice, both wild-type and ΔTRP120 *E. japonica* multiplied to high levels in various tissues, with similar clinical chemistry and hematologic changes, proinflammatory cytokine induction, and fatal disease. However, the blood levels of ΔTRP120 *E. japonica* were almost undetectable within 24 h, whereas the levels of the wild type increased exponentially. Greater than 90% of TRP120 was released from infected cells into the culture medium. Mouse blood monocytes exposed to native TRP120 from culture supernatants showed significantly reduced cell surface expression of the transmigration-related markers Ly6C and CD11b. Larval ticks attached to mice infected with either wild-type or ΔTRP120 *E. japonica* imbibed similar amounts of blood and subsequently molted to nymphs at similar rates. However, unlike wild-type *E. japonica*, the ΔTRP120 mutant was minimally acquired by larval ticks and subsequent molted nymphs and, thus, failed to transmit to naïve mice. Thus, TRP120 is required for bacteremia but not disease. These findings suggest a novel mechanism whereby an obligatory intracellular bacterium manipulates infected blood monocytes to sustain the tick–mammal transmission cycle.

**IMPORTANCE:**

Effective prevention of tick-borne diseases such as human ehrlichiosis requires an understanding of how disease-causing organisms are acquired. *Ehrlichia* species are intracellular bacteria that require infection of both mammals and ticks, involving cycles of transmission between them. Mouse models of ehrlichiosis and tick–mouse transmission can advance our fundamental understanding of the pathogenesis and prevention of ehrlichiosis. Herein, a mutant of *Ehrlichia japonica* was used to investigate the role of a single *Ehrlichia* factor, named tandem repeat protein 120 (TRP120), in infection of mammalian and tick cells in culture, infection and disease progression in mice, and tick acquisition of *E. japonica* from infected mice. Our results suggest that TRP120 is necessary only for *Ehrlichia* proliferation in circulating mouse blood and ongoing bacteremia to permit *Ehrlichia* acquisition by ticks. This study provides new insights into the importance of bacterial factors in regulating bacteremia, which may facilitate tick acquisition of pathogens.

## INTRODUCTION

Based on pathophysiological similarity to canine monocytic ehrlichiosis and immunological cross-reactivity and ultrastructural similarity to *Ehrlichia canis*, the first human-pathogenic *Ehrlichia* species in the US, later named as *Ehrlichia chaffeensis,* was discovered in 1986 (1–3). Since then, new human-pathogenic *Ehrlichia* species/subspecies/strains have been continuously discovered (4–7). Among human-pathogenic *Ehrlichia* species, *E. chaffeensis* infection is most prevalent. *E. chaffeensis* causes human monocytic ehrlichiosis (HME), which is a severe flu-like febrile illness with signs of hepatitis that oftentimes requires hospitalization (40%–63% of cases) (8). Although doxycycline is generally effective in treating HME (9), no alternatives exist should microbial resistance develop. No effective vaccine is currently available for any ehrlichiosis of humans or animals.

Ticks acquire ehrlichiae by biting infected vertebrates. Then, ehrlichiae are transstadially transmitted through molting to the next developmental stage of ticks (larva → nymph → adult) prior to transmission to the next vertebrate host (10). *E. canis* and *E. chaffeensis*, however, cannot be transmitted vertically (transovarially) in ticks (11, 12), and thus, ehrlichiae depend on cyclical transmission and infection between ticks and mammals to exist in nature. *E. chaffeensis* can be directly transmitted between human individuals via blood transfusion or tissue transplantation (13), but the primary mode of human infection is the bite of ticks that are infected by parasitizing infected animals. *Ehrlichia* sp. HF, which is also called *Ixodes ovatus Ehrlichia* (IOE) agent and officially classified as *Ehrlichia japonica* recently (14), was originally isolated from *I. ovatus* ticks but also found in rodents and dogs in Japan (15–17). *E. japonica* has a relatively broad geographic distribution and several tick vectors, and indeed, it has been found in *Ixodes ricinus* and *Ixodes apronophorus* ticks in several European countries (18–20). *E. japonica* is evolutionally closest to *Ehrlichia muris* followed by *E. chaffeensis,* whereas *E. chaffeensis* is closest to *E. japonica* (16, 21). Laboratory mice infected with *E. japonica* experience progressive infection that may lead to subclinical to fatal disease in a dose-dependent manner [median lethal dose (LD_50_) = 100 bacteria] (22), and the immunopathogenesis of which has been studied extensively (16, 23–29). As such, *E. japonica* could serve as a valuable component of a small-animal disease model of human ehrlichiosis and tick transmission.

We recently reported the successful cultivation of *E. japonica* in a DH82 canine histiocytic leukemia cell line and the subsequent achievement of whole-genome sequencing (21) and construction of a Himar1 transposon mutant library comprising at least 158 distinct mutants (22). Among the Himar1 transposon mutants is *E. japonica* EHF_0993 (*TRP120*)::Himar1 encoding an inserted mutant of tandem repeat protein 120 (TRP120) (22). Homologs of TRP120 that have four or more tandem intramolecular repeats are universally found in all sequenced *Ehrlichia* species (21). Based on Blast analysis, the expected *E*-value between *E. chaffeensis* and *E. japonica* TRP120 is 2 × *e*^–44^, which is considered to reflect the high similarity of protein homologs between these two organisms (30). *E. chaffeensis* Arkansas TRP120 is an effector of the type 1 secretion system and interacts with over one dozen diverse mammalian proteins and host nuclear DNA and has various activities, such as facilitating pathogen entry into host cells, serving as a nucleomodulin, and regulating signal transduction and apoptosis (31–39).

In the present study, we first cloned and characterized the *E. japonica TRP120*::Himar1 mutant (ΔTRP120). We then investigated its ability to infect cultures of mammalian cells or tick cells, its colonization of various organs and tissues, and the details of its pathogenesis in mice. In addition, we used a newly developed *Ixodes* tick transmission model to examine the transmission of *E. japonica* wild type (WT) and ΔTRP120 between mammals and ticks. Our results indicate that TRP120 is the first example of an *Ehrlichia* factor that is required for tick acquisition, independently of mammalian or tick cell infection and *in vivo* pathogenesis.

## RESULTS

### *Ehrlichia japonica* ΔTRP120 does not affect the proliferation in mammalian and tick cell cultures

**Fig 1:**
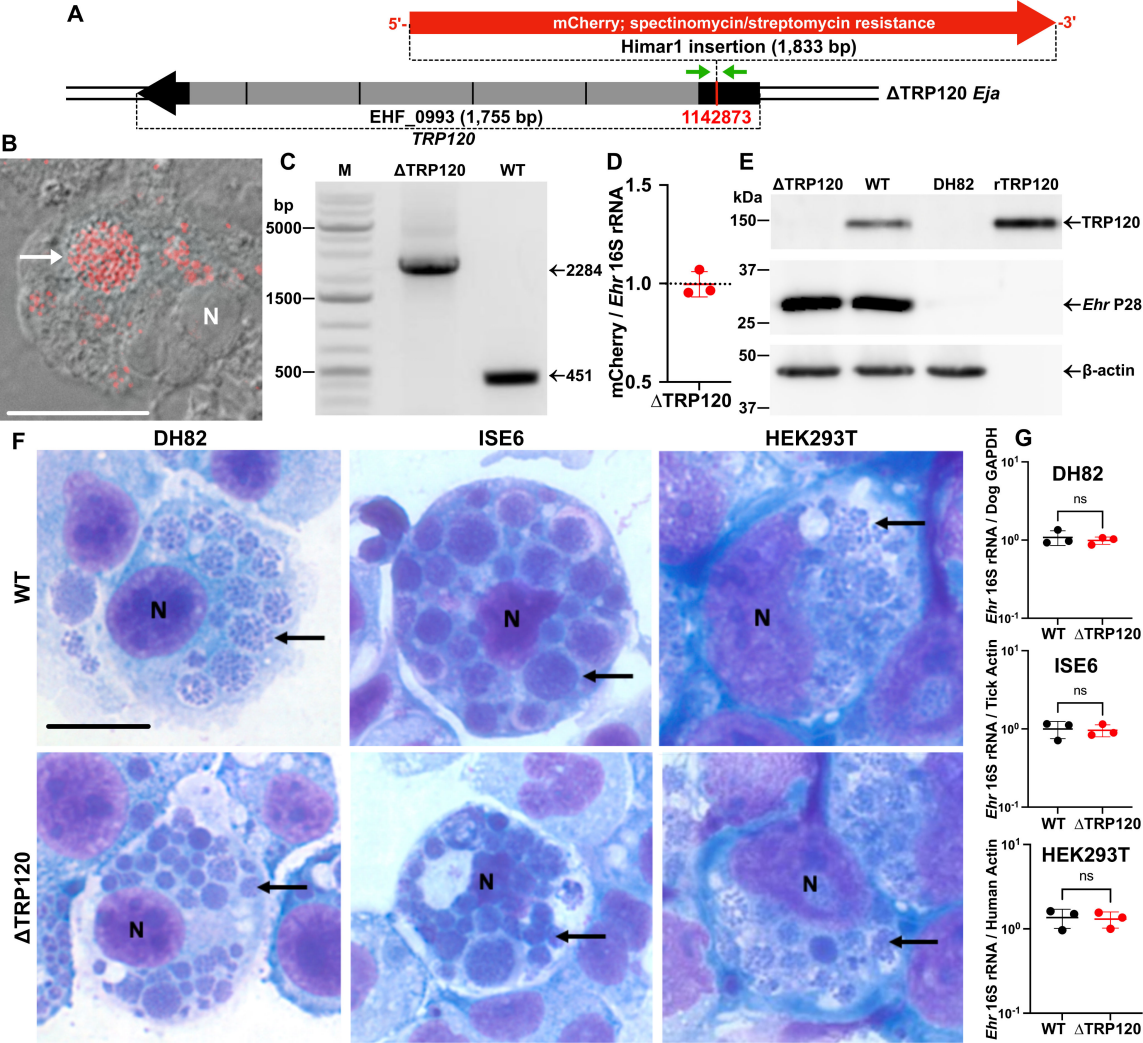
Cloned ΔTRP120 does not express TRP120 but can infect and replicate in mammalian and tick cells in culture similarly to WT *E. japonica*. (**A**) Himar1 transposon insertion site of mCherry and spectinomycin/streptomycin resistance genes (selection markers) of *TRP120*. The insertion site was mapped at the genomic locus at 1,142,873, which is near the 5′-end of *TRP120* upstream of the 300-bp tandem repeats (gray boxes). (**B**) ΔTRP120 expressing mCherry fluorescence (white arrow) in DH82 macrophages. The fluorescence/DIC merged image was acquired using DeltaVision microscopy. (**C**) Himar1 transposon insertion site-specific flanking PCR to verify mutant clonality. Primer locations were indicated with green arrows in panel (A). M, DNA size markers. PCR target size for WT or other Himar1 mutants of *E. japonica*, 451 bp; for ΔTRP120 with the Himar1 insertion sequence (1,833 bp), 2,284 bp. (**D**) The ratio of copy numbers of mCherry vs *Ehrlichia* (*Ehr*) 16S rRNA. DNA samples were purified from DH82 cells infected with the cloned ΔTRP120, and qPCR was performed with primers for mCherry and *Ehrlichia* 16S rRNA gene, using pCis-mCherry-SS-Himar-A7 and *Ehrlichia* 16S rDNA cloned in pUC19 plasmids as standards. (**E**) TRP120 is expressed by WT but not ΔTRP120. Cell lysates of uninfected and WT or ΔTRP120-infected DH82 cells were subjected to western blotting with mouse anti-rTRP120 serum. The extent of infection with WT or ΔTRP120 was assessed with rabbit anti-*Ehrlichia* P28, and sample input was normalized by β-actin. (**F**) Mammalian cells (DH82 or HEK293T) or tick cells (ISE6) in culture were infected with WT or ΔTRP120. ΔTRP120 did not affect the extent of infection or bacterial replication (examples of bacteria-containing morulae indicated by black arrows) compared to WT, as assessed with HEMA 3 staining. (**B, F**) N, nucleus. Scale bar, 10 µm. (**G**) Quantitation of bacterial infection/host cells by RT-qPCR. Ct values of *Ehrlichia* 16S rRNA were normalized by the respective host genes. ns, not significantly different by Student’s *t*-test (*N* = 3, *P* < 0.05).

*E. japonica* HF TRP120 comprises 584 amino acids [AA, calculated molecular weight (MW) of 62,809 Da] encoded by EHF_0993 (1,755 bp) and contains tandem four complete 100-residue repeats and one partial 25-residue repeat (Fig. 1A) (21). In *TRP120*::Himar1 mutant, the 1.8-kb Himar1 sequence containing mCherry and the selection markers for spectinomycin/streptomycin resistance was mapped at genomic locus 1,142,873, which was at the upstream of the repeats (near the 5′-end) of *TRP120* gene (Fig. 1A) (22). To characterize this mutant, monoclonal *TRP120*::Himar1 mutant was obtained by a repeated limited dilution of the original culture H60-E2 containing *TRP120*::Himar1 mutant (22). Cloned *TRP120*::Himar1 (ΔTRP120) was selected by mCherry fluorescence and verified by insertion site-specific “flanking PCR” for *TRP120* gene (22), with ΔTRP120 showing a single larger PCR product containing the Himar1 insert (Fig. 1A through C). Himar1 insertions occur mostly once per genome and rarely twice (40, 41). To verify that there are no additional Himar1 insertions in the genome of ΔTRP120 mutant, quantitative PCR (qPCR) was performed, and the results showed an approximate ratio of 1:1 for the copy numbers of the mCherry gene vs the single-copy *Ehrlichia* 16S rRNA gene (Fig. 1D), indicating a single Himar1 transposon insertion per *Ehrlichia* genome. In ΔTRP120 *E. japonica*, TRP120 protein expression was absent, as demonstrated by western blotting using mouse antisera developed against full-length recombinant TRP120 (rTRP120), whereas *Ehrlichia* major outer membrane protein P28 (42) was expressed as WT *E. japonica* (Fig. 1E). Both native TRP120 expressed by WT and rTRP120 were detected at ~150 kDa (Fig. 1E), which is much larger than the predicted MW (63 kDa) but similar to what was reported for *E. chaffeensis* Arkansas TRP120 (43). This is unlikely due to the post-translational modifications of *E. japonica* TRP120 such as glycosylation, SUMOylation, or phosphorylation of Ser/Thr/Tyr as rTRP120 was purified from *Escherichia coli*. Rather, this is probably due to the presence of a large percentage of acidic AA (44) as *E. japonica* TRP120 contains 110 strongly acidic AA (D and E; 19%) and 181 polar AA (S, T, Y, N, Q, and C; 31%) with pI of 4.2. Both ΔTRP120 and WT replicated similarly in DH82 (canine macrophage line), HEK293T (human embryonic kidney line), and ISE6 (*Ixodes scapularis* tick embryonic cell line), with more than 10 large bacteria-containing morulae (intracellular microcolonies of bacteria) filling the cytoplasm (Fig. 1F) and similar bacterial load by reverse transcription-qPCR (RT-qPCR) (Fig. 1G).

### TRP120 is not essential for infecting mice, eliciting disease, and inducing a set of cytokines

**Fig 2:**
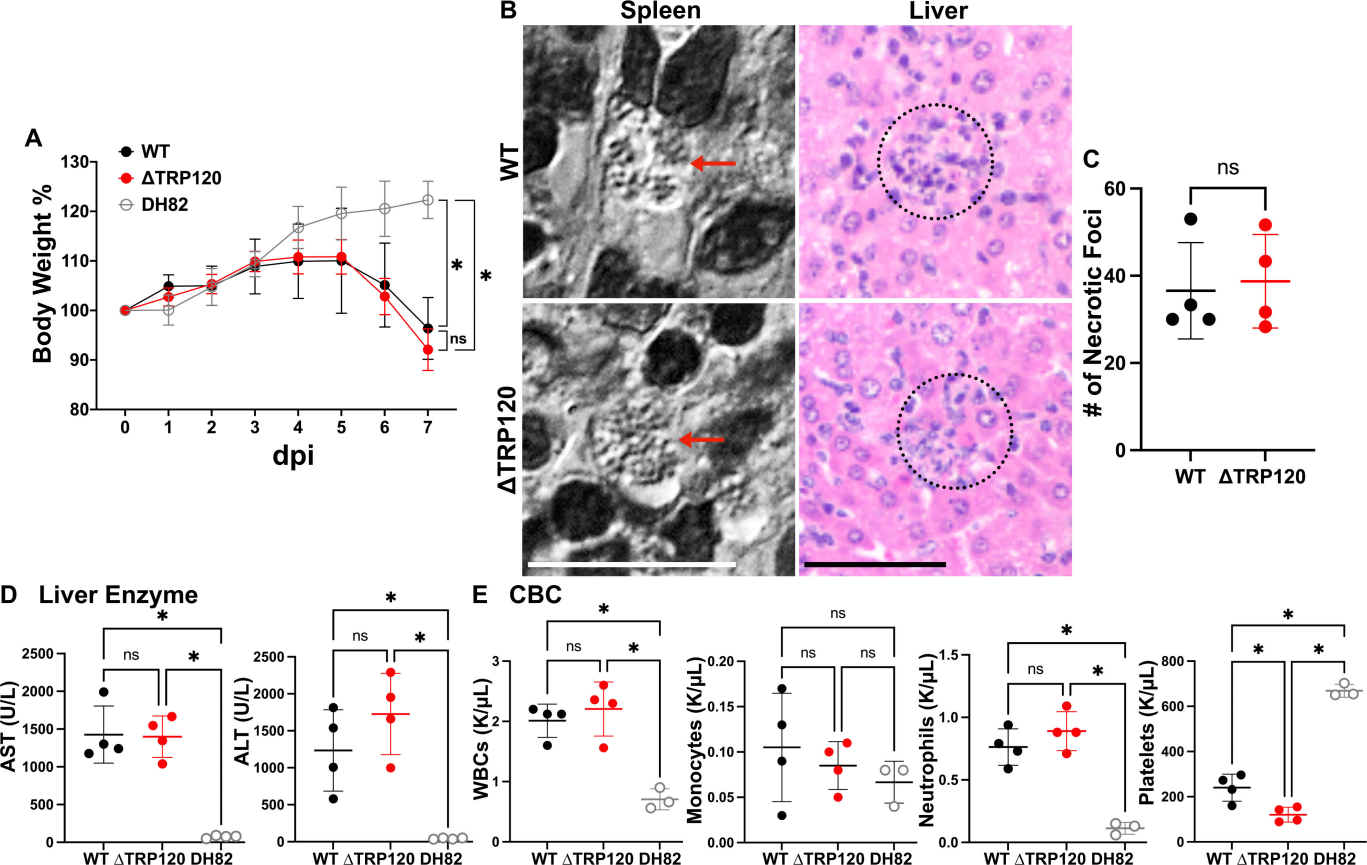
Lack of TRP120 does not alter *E. japonica* pathogenesis in mice. (**A**) Temporal change in body weight of mice inoculated with WT or ΔTRP120-infected DH82 cells. Uninfected DH82 cells served as the control. Mice in each experimental group experienced similar acute body weight loss after day 5 d pi. ^*^significantly different (*P <* 0.05); ns, not significantly different by repeated-measures analysis of variance. (**B**) Left: *E. japonica* morulae with visible individual bacteria (red arrows) in the cytoplasm of macrophages in the spleen of both WT and ΔTRP120–infected mice at 7 d pi. Differential interference contrast. Scale bar, 10 μm. Right: necrotic/apoptotic hepatocytes with pyknotic nuclei and inflammatory cell aggregates (dashed circle) in the liver of both WT and ΔTRP120-infected mice at 7 d pi (hematoxylin and eosin staining). Scale bar, 50 μm. (**C**) Quantitation of necrotic foci/6 mm^2^ of liver sections each from WT and ΔTRP120-infected mice. ns, not significantly different by Student’s *t*-test. (**D, E**) Serum biochemistry for liver function profiling (**D**) and automated complete blood counting (CBC; K/μL, cell numbers in thousands per microliter) of white blood cells (WBCs), monocytes, neutrophils, and platelets (**E**) of WT or ΔTRP120-infected or uninfected mice at 7 d pi. ns, not significantly different; ^*^significantly different (*P <* 0.05) by analysis of variance. *Ehrlichia* inoculum per mouse was determined by qPCR: (**A, D, E**) WT, 801; ∆TRP120, 850. (**B, C**) WT, 1,450; ∆TRP120, 1,035.

We next investigated whether the infection with *E. japonica* and ehrlichiosis onset in immunocompetent mice require TRP120. Mice were intraperitoneally (ip) inoculated (~10 cells per mouse) with uninfected or infected DH82 cells containing approximately 1,000–2,000 ΔTRP120 or WT. Both groups of mice inoculated with ΔTRP120 or WT lost body weight rapidly (Fig. 2A) and showed progressively severe clinical signs (squinty eyes, hunched back, ruffled fur, and inactivity) starting 5 days post-inoculation (d pi) and required euthanasia at 7 d pi. The control mice inoculated with uninfected DH82 cells gained body weight continuously and did not show any clinical signs (Fig. 2A). Histopathologic examination of mice inoculated with ΔTRP120 or WT revealed similar lesions, i.e., necrotic/apoptotic hepatocytes with pyknotic nuclei and inflammatory cell aggregates in liver samples (Fig. 2B and C), and *E. japonica* morulae were observed in spleen samples (Fig. 2B) with similar bacterial loads confirmed by qPCR (Fig. 4B) at 7 d pi. Both groups of mice inoculated with ΔTRP120 or WT showed similar severe signs of liver injury, i.e., high levels of serum alanine aminotransferase (ALT) and aspartate aminotransferase (AST) compared with uninfected mice (Fig. 2D). Compared with uninfected mice, both groups of infected mice showed severe thrombocytopenia, leukocytosis, and neutrophilia, but monocyte numbers were not significantly different (Fig. 2E). Between two groups of infected mice, except for significantly lower platelet counts in ΔTRP120 than WT-inoculated mice, there were no significant differences on white blood cell, monocyte, and neutrophil counts (Fig. 2E).

**Fig 3:**
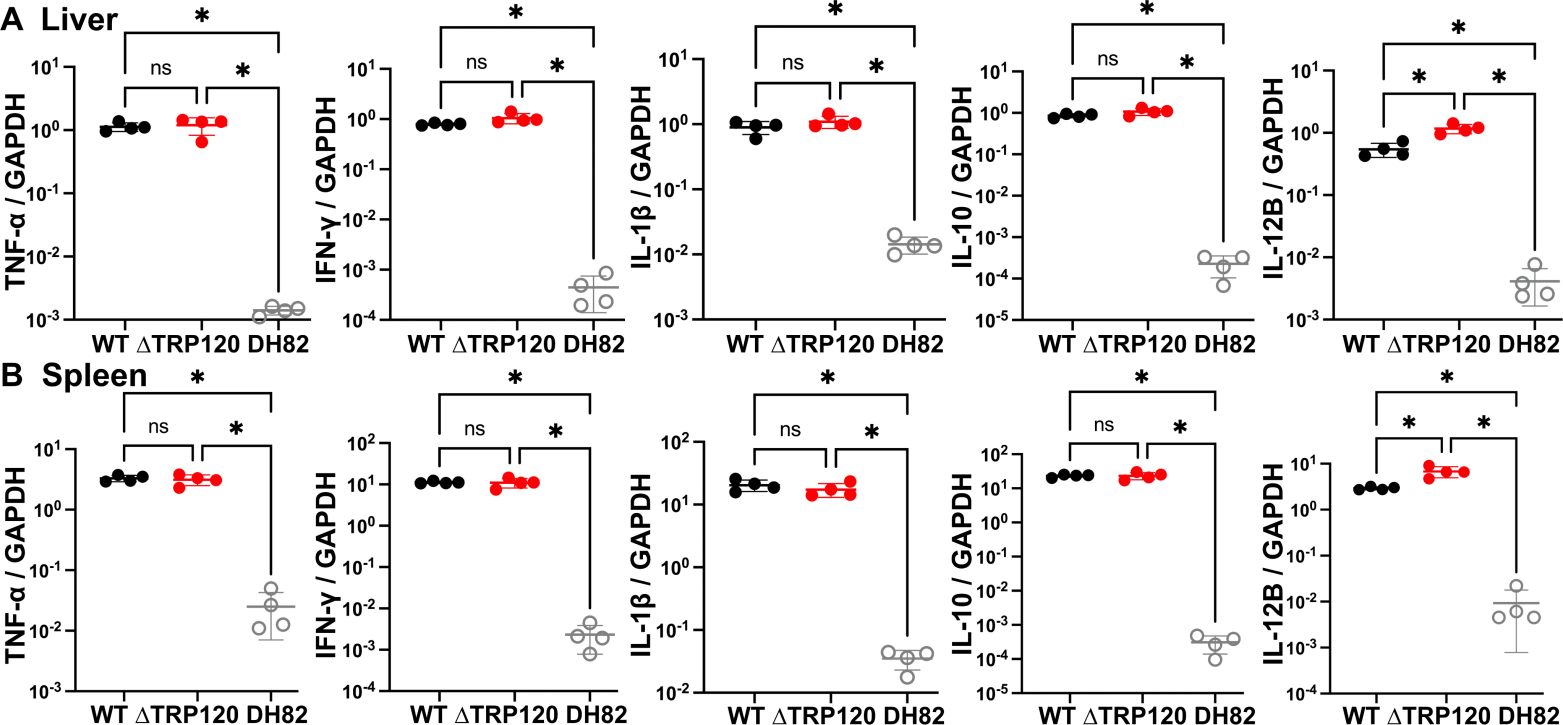
Similar inflammatory cytokine mRNA activation in mice challenged with WT or ΔTRP120 *E. japonica*. Levels of selected cytokine mRNAs in the liver (**A**) and spleen (**B**) of mice inoculated with WT or ΔTRP120-infected DH82 macrophages were determined at 7 d pi by RT-qPCR normalized against those of mouse glyceraldehyde 3-phosphate dehydrogenase (GAPDH) mRNA. *Ehrlichia* inoculum per mouse (WT, 801; ∆TRP120, 850) was determined by qPCR. Uninfected DH82 cells served as the negative control. Data are presented as the mean ± SD (*N* = 4) and are representative of three independent experiments. ns, not significantly different; ^*^significantly different by Student’s *t*-test (*P* < 0.05).

Cells in the liver of mice infected with WT *E. japonica* undergo activation of genes encoding the cytokines TNF-α, IL-1β, IL-6, IFN-γ, IL-10, and IL-12p40 (22, 29). In both the liver and spleen of ΔTRP120 and WT*-*infected mice, significant (~100–10,000-fold compared with DH82 cell control) induction of the cytokines TNF-α, IFN-γ, IL-1β, IL-10, and IL-12p40 (IL-12B) mRNA occurred. However, between ΔTRP120 and WT infection, in both the liver and spleen, no significant difference was found, with the exception of a small but significant increase in IL-12p40 mRNA level with ΔTRP120 (Fig. 3). Thus, lack of TRP120 did not seriously alter *E. japonica* clinical signs, liver injury, leukocyte abnormality, and induction of the set of cytokine gene expression in mice.

### *Ehrlichia japonica* ΔTRP120 can spread and proliferate in major tissues but cannot be retained in the blood of mice

**Fig 4:**
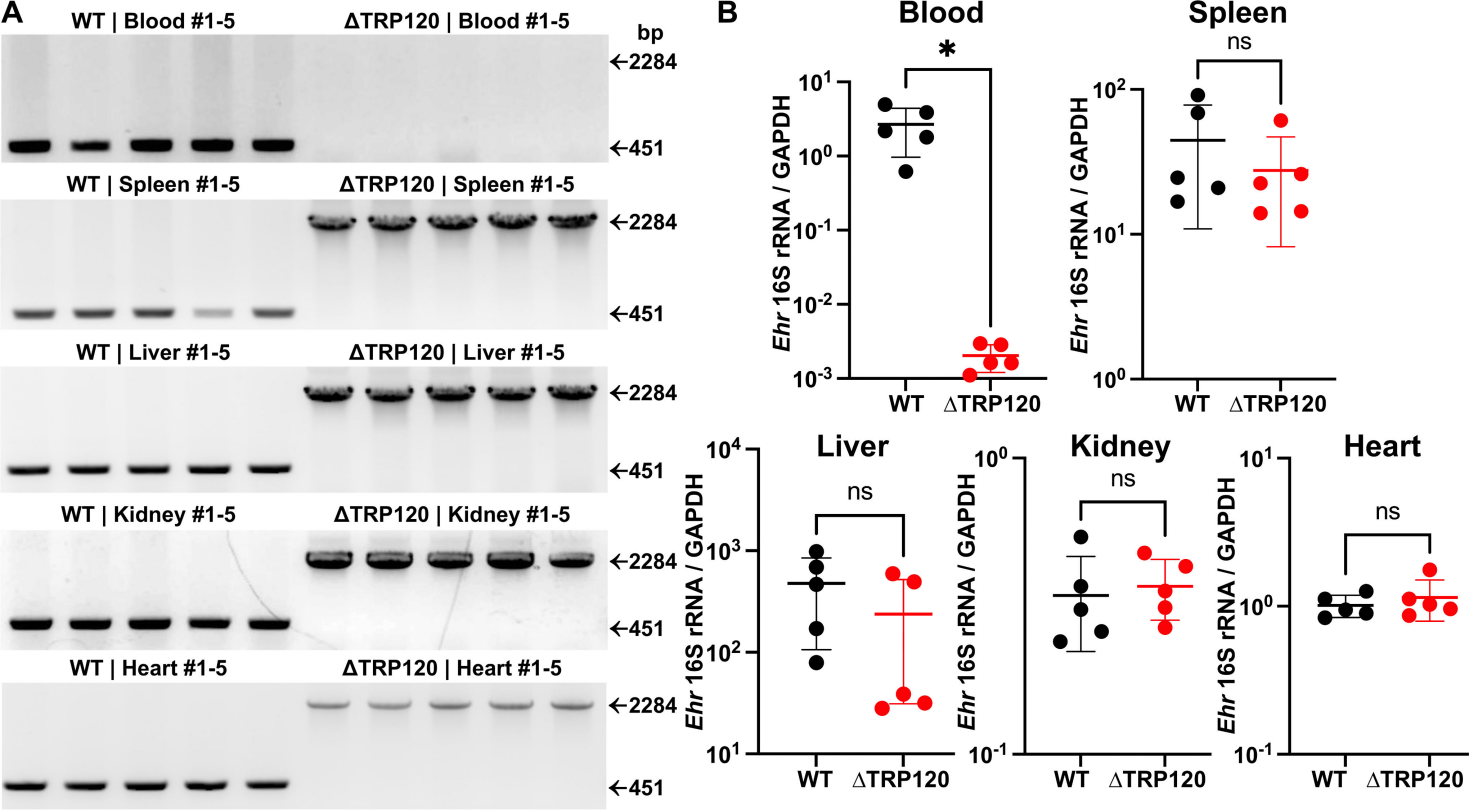
Similar levels of WT or ΔTRP120 are found in all tissues of infected mice except in the blood. (**A**) Himar1 insertion site-specific flanking PCR confirming infection of WT (amplicon size, 451 bp) and ΔTRP120 (amplicon, 2,284 bp) *E. japonica* in blood, spleen, liver, kidney, and heart of individual mice as assessed at 7 d pi (ip, *N* = 5). *Ehrlichia* inoculum per mouse (WT, 1,450; ∆TRP120, 1,035) was determined by qPCR. (**B**) Bacterial loads in each sample were determined by qPCR based on *Ehrlichia* 16S rRNA gene and normalized against the level of mouse *GAPDH*. Data are presented as the mean ± SD (*N* = 5) and are representative of three independent experiments (*Ehrlichia* inoculum per mouse: Experiment 1: WT, 1,450; ∆TRP120, 1,035. Experiment 2: WT, 801; ∆TRP120, 850. Experiment 3: WT, 2,385; ∆TRP120, 3,218). ns, not significantly different; ^*^significantly different by Student’s *t*-test (*P* < 0.05).

To investigate the role of TRP120 in the infection of *E. japonica* in tissues/organs, mice were ip injected with WT or ΔTRP120. The clonality of ΔTRP120 in the organ and tissue of each individual mouse was analyzed by flanking PCR at 7 d pi to ensure that the observed similarities in clinical signs and pathophysiology were not due to any reversion of ΔTRP120 to WT. Reversion was not observed, indicating that the ΔTRP120 clone remained stable in mice (Fig. 4A). Strikingly, ΔTRP120 was conspicuously absent in the blood of every mouse despite its presence in the spleen, liver, kidney, and heart (Fig. 4A). In contrast, WT infection was clearly detected in the blood as well as other tissues (Fig. 4A). To verify the results of the flanking PCR analysis, bacterial load in the blood and other tissues of ΔTRP120 or WT-infected mice was quantified by qPCR specific to *Ehrlichia* 16S rRNA gene. The results confirmed that while bacterial loads in the spleen, liver, kidney, and heart were not significantly different between ΔTRP120 and WT-infected mice, *E. japonica* was almost undetectable in the blood of every ΔTRP120-infected mouse; by comparison, WT was present at a >1,000-fold higher level in the blood (Fig. 4B). In both groups of mice, bacterial loads were greater in the spleen and liver than in the heart and kidney when normalized to the level of mouse *GAPDH* gene (Fig. 4B). These results suggested that TRP120 is essential for bacteremia.

**Fig 5:**
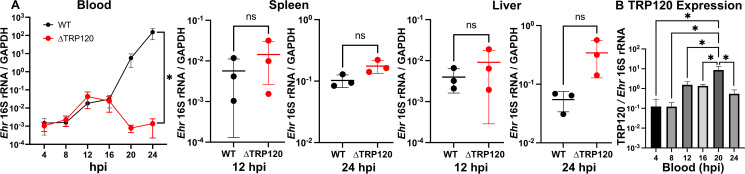
ΔTRP120-infected blood monocytes rapidly exit blood vessels and enter tissues. (**A**) Mice were iv inoculated with high doses (~5 × 10^8^) of host cell-free WT or ∆TRP120, and bacterial loads were assessed in blood from 4 to 24 h pi and in the spleen and liver at 12 and 24 h pi. Bacterial loads were determined by RT-qPCR based on *Ehrlichia* 16S rRNA normalized against the level of mouse GAPDH mRNA. (**B**) RT-qPCR showing *TRP120* expression in WT in blood samples at 4–24 h pi normalized by *Ehrlichia* 16S rRNA gene. *Ehrlichia* inoculum per mouse (WT, 5.04 × 10^8^; ∆TRP120, 4.58 × 10^8^) was determined by qPCR. Data are presented as the mean ± SD (*N* = 3) and are representative of three independent experiments. ns, not significantly different by Student’s *t-*test; ^*^significantly different by repeated-measures analysis of variance (*P <* 0.05).

*E. japonica* cannot survive and replicate outside of host cells, which are monocytes and tissue macrophages in mice, and ehrlichiae are non-motile (lack flagella) (21). ΔTRP120 was undetectable in blood at 7 d pi, yet levels comparable to WT were found in other tissues, suggesting that ΔTRP120-infected monocytes were present in circulating blood, having extravasated into surrounding tissues and differentiated into macrophages earlier than 7 d pi. To investigate this possibility directly, mice were intravenously (iv) inoculated with freshly prepared host cell-free ΔTRP120 or WT, instead of ip inoculation of *E. japonica*-infected DH82 cells that requires extra steps for *Ehrlichia* to disseminate and infect mouse blood monocytes. The early time course of both ΔTRP120 and WT infection was assessed by RT-qPCR quantification of blood samples collected every 4 h pi till 24 h pi and of spleen and liver samples collected at 12 and 24 h pi. RT-qPCR was used rather than qPCR to detect live bacteria, i.e., to exclude the detection of DNA from dead bacteria. Up to 12 h pi, both ΔTRP120 and WT increased exponentially in blood, implying replication in monocytes (Fig. 5A). Strikingly, starting at 12–16 h pi, the amount of ΔTRP120 rapidly decreased from the blood, whereas WT continued exponential growth in blood monocytes (Fig. 5A). On the other hand, levels of ΔTRP120 and WT in the liver and spleen at 12 and 24 h pi did not differ significantly, although ΔTRP120 tended to be more abundant than WT in both tissues at 12 and 24 h pi (Fig. 5A). Both ΔTRP120 and WT increased by almost 10-fold in the spleen and liver from 12 to 24 h pi (Fig. 5A). Thus, monocytes supported ΔTRP120 replication in the blood up to 12–16 h pi but rapidly extravasated and spread to the liver and spleen around 12 h pi. In WT, TRP120 mRNA was upregulated by approximately 10-fold in the blood starting at 12 h pi (iv) (Fig. 5B), which coincided with the divergence of WT-infected monocytes from ΔTRP120-infected monocytes in the blood (Fig. 5A). These results implied that TRP120 is essential for the continuous replication of *Ehrlichia* in monocytes and retention of infected monocytes in the blood, as *Ehrlichia-*infected monocytes lacking TRP120 were largely absent from blood after 20 h pi (iv).

### TRP120 protein is released from infected host cells and suppresses expression of extravasation-related monocyte surface markers

**Fig 6:**
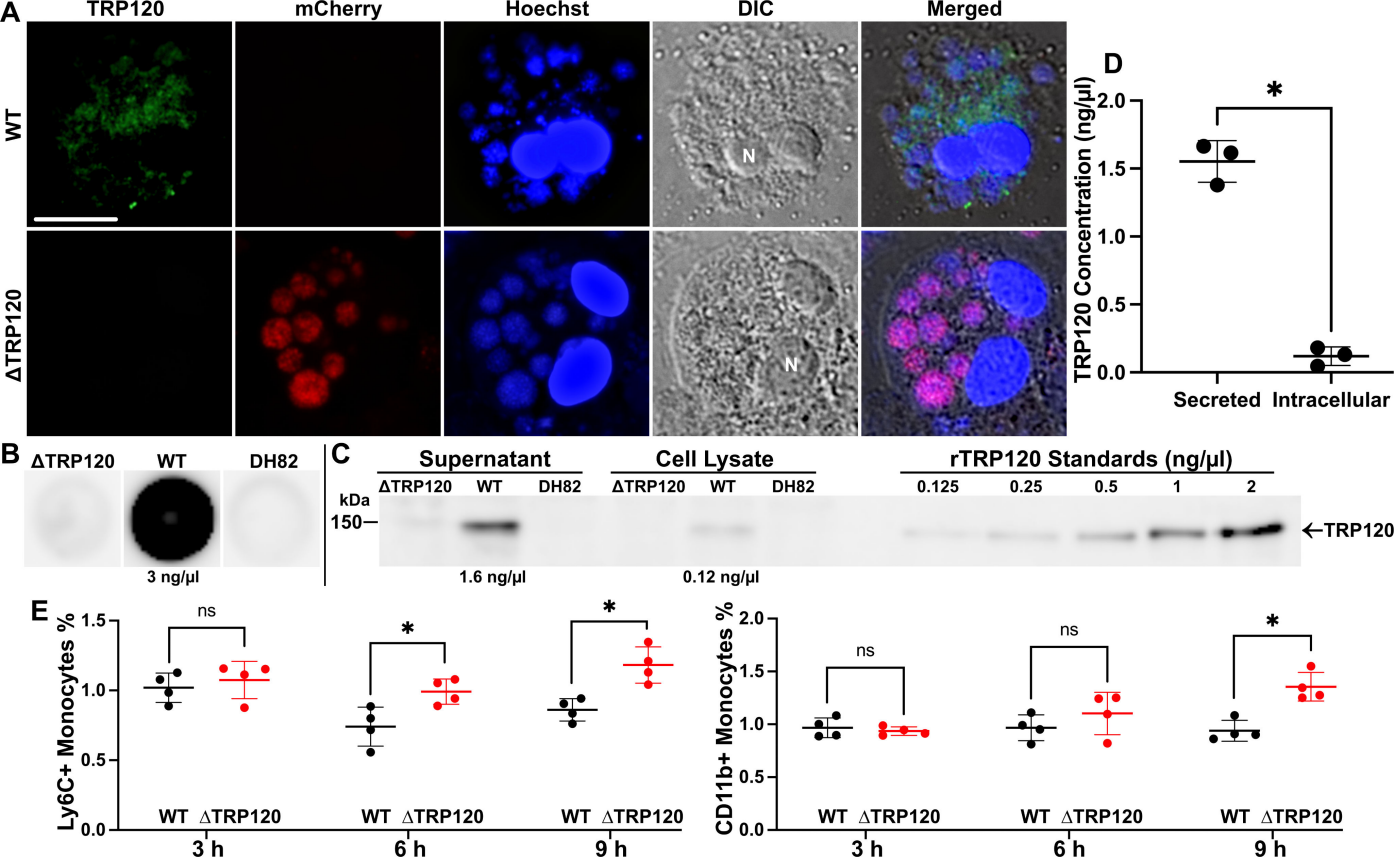
Majority of TRP120 is released from *E. japonica*-infected DH82 cells to the culture medium, which suppresses Ly6C and CD11b expression on mouse peripheral blood monocytes. (**A**) Immunofluorescence labeling of DH82 macrophages infected with WT or ΔTRP120 (mCherry-expressing) *E. japonica* at 3 d pi with mouse anti-rTRP120 serum and AF488-conjugated anti-mouse IgG (green). *E. japonica* and host DNAs were labeled with Hoechst 33342 (blue). DIC, differential interference contrast. Scale bar, 10 μm. (**B**) Dot-immunoblotting results showing native TRP120 was secreted into the culture supernatant from 10^6^ *E. japonica*-infected DH82 cells (estimated ~3 ng/µL) but not with ΔTRP120. Uninfected DH82 cells served as the negative control. (**C**) Western blot analysis of culture supernatant and cell lysates of 10^6^ DH82 cells infected with WT or ΔTRP120. Recombinant TRP120 proteins were serially diluted to create a standard curve for densitometry quantitation using ImageJ. Approximately 1.6 ng/µL TRP120 was secreted from WT (supernatant) vs ~0.12 ng/µL that was intracellular (cell lysate). (**D**) Statistical analysis of western blot data for secreted vs intracellular TRP120 concentrations determined by densitometry from three independent cultures. Data are presented as the mean ± SD (*N* = 3); ^*^significantly different by Student’s *t*-test (*P* < 0.001). (**E**) Flow cytometry results showing Ly6C and CD11b expression of mouse peripheral blood mononuclear cells (PBMCs) incubated with the culture supernatant of ∆TRP120 and WT-infected DH82 cells for 3, 6, and 9 h. Data were normalized with PBMC incubated with an uninfected DH82 cell culture supernatant. Data are from four independent experiments and presented as the mean ± SD; ns, not significantly different; ^*^significantly different by Student’s *t*-test (*P* < 0.001).

Given that *E. japonica* TRP120 and *E. chaffeensis* Arkansas TRP120 are predicted to have similar C-terminal T1SS signals (45), we determined the subcellular localization of native TRP120 in *E. japonica*-infected cells by immunofluorescence labeling with a TRP120-specific antibody, revealing that TRP120 was distributed at both inside and outside of WT morulae in the cytoplasm of infected cells (Fig. 6A). TRP120 was not detected in ΔTRP120-infected DH82 cells (Fig. 6A), confirming the western blotting results (Fig. 1E). Because the distribution of TRP120 was rather diffuse, we also harvested the culture supernatant from WT and ΔTRP120-infected DH82 cells to assess whether TRP120 was released from WT-infected DH82 cells. Using a standard curve based on serial-diluted rTRP120, dot-immunoblot analysis of the non-denatured protein detected ~3 ng/µL of native TRP120 from culture supernatant of 10^6^ infected DH82 cells at 2 d pi (Fig. 6B). Similarly, western blot analysis of denatured protein detected ~1.6 ng/µL of native TRP120 in culture supernatant and ~0.12 ng/µL in cell lysates (intracellular content from 10^6^ infected DH82 cells in 5 mL culture) (Fig. 6C and D). This indicated that >90% of TRP120 was released from the infected host cells into the culture supernatant.

To study how TRP120 regulates the retention of *E. japonica*-infected monocytes in the blood, we examined two well-known extravasation-related monocyte surface markers: Ly6C (lymphocyte antigen 6 complex, locus C1) and CD11b (Mac-1 integrin). Monocytes expressing high levels of Ly6C (Ly6C^H^ monocytes) can effectively traverse from the blood vessel lumen to infiltrate inflammatory sites (46, 47). Ly6C^H^ monocytes express low levels of CX_3_CR1 chemokine receptor CXCL1/IL8 (CX_3_CR1^low^Ly6C^high^), whereas CX_3_CR1^high^Ly6C^low^-displaying monocytes (Ly6C^L^ monocytes) are long-lived in the bloodstream. CD11b regulates leukocyte adhesion and migration to mediate the inflammatory response and also attenuates proinflammatory responses (48). Mouse PBMCs were incubated with the freshly harvested culture supernatants of WT (containing native TRP120), ΔTRP120 mutant*,* and uninfected DH82 cells (as negative control for data normalization, as WT and ΔTRP120 were cultured in DH82 cells), and the time course of Ly6C and CD11b expression was determined by flow cytometry. As shown in Fig. 6E, after 6 and/or 9 h post-incubation, PBMCs incubated with the culture supernatant of ΔTRP120 increased Ly6C and CD11b expression compared with those incubated with the culture supernatant of WT. This suggests that TRP120 is required to suppress Ly6C and CD11b upregulation on mouse peripheral blood monocytes.

### Diminished bacteremia of ΔTRP120 can be transiently restored via transfection of infected cells with a plasmid encoding rTRP120

**Fig 7:**
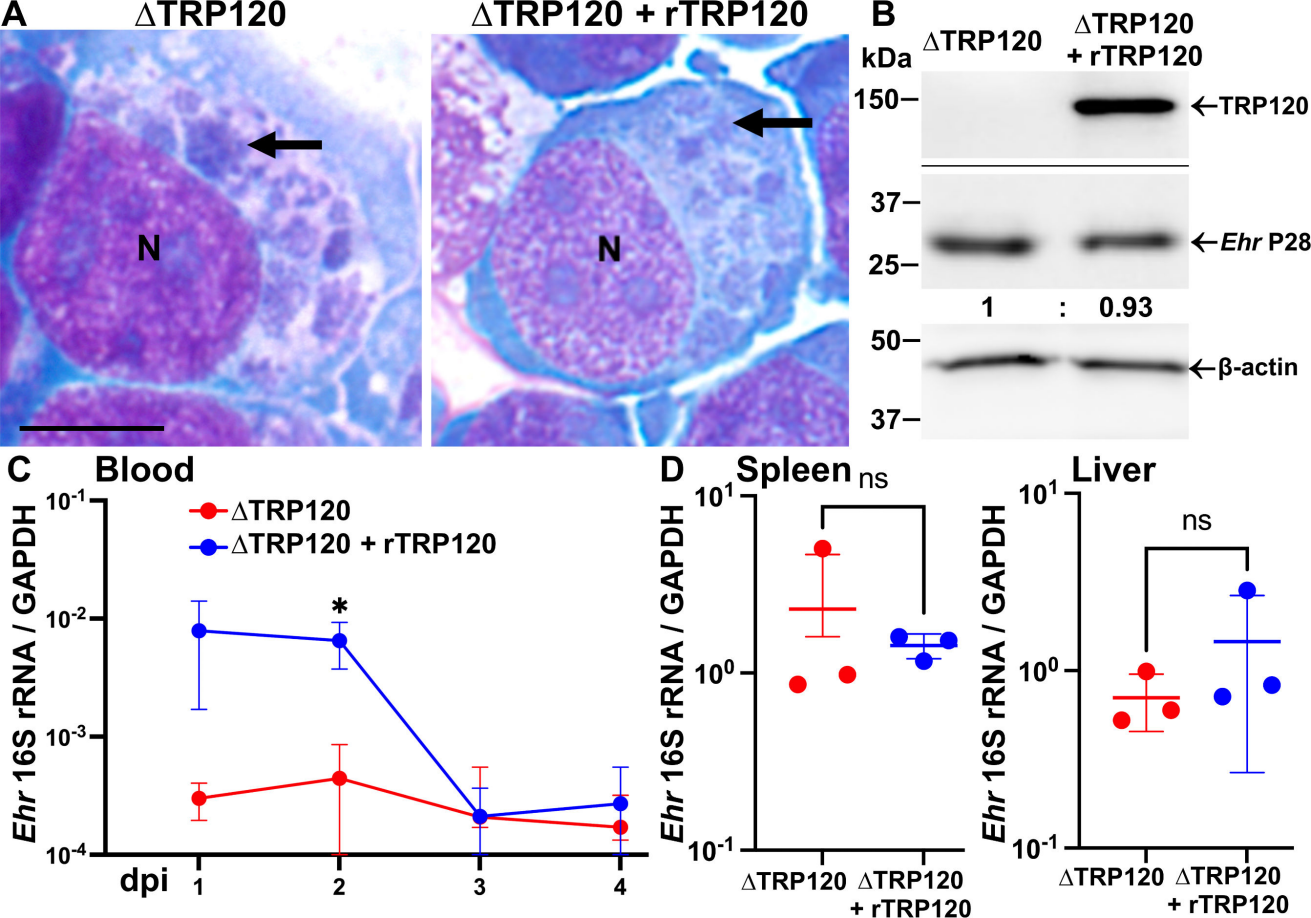
Reduced bacteremia of ΔTRP120 *E. japonica* is partially restored by the ectopic expression of TRP120. (**A, B**) HEK293T cells infected with ΔTRP120 *E. japonica* (arrow) with or without transfection of FLAG-TRP120 (rTRP120) at 1 d pi. (**A**) HEMA 3 staining. Scale bar, 10 μm. (**B**) Western blotting using anti-FLAG, *Ehrlichia* (*Ehr*) P28, and β-actin. Relative ratios (*Ehr* P28/β-actin) of western blotting band intensities were determined by ImageJ, with the ratio of non-transfected ΔTRP120 set as 1. (**C, D**) Mice were ip inoculated with HEK293T cells infected with ΔTRP120 with or without transfection of FLAG-TRP120. qPCR analysis was performed using *Ehrlichia* 16S rRNA normalized by mouse GAPDH. (**C**) Temporal bacterial loads in mouse blood. ^*^Significantly different by two-way repeated-measures analysis of variance (*P* < 0.05). (**D**) Spleen and liver. *Ehrlichia* inoculum per mouse (∆TRP120, 2.36 × 10^7^; ∆TRP120 + rTRP120 = 1.10 × 10^7^) was determined by qPCR. Data are presented as the mean ± SD (*N* = 3) and are representative of two independent experiments. ns, not significantly different by Student’s *t*-test.

For ehrlichiae, molecular complementation is difficult due to extremely inefficient homologous recombination and selection of the complemented mutant. We, therefore, attempted to mimic TRP120 secretion from infected host cells by ectopically expressing TRP120 in ΔTRP120-infected host cells and examined functional complementation of diminished bacteremia of ΔTRP120 in mice. For this purpose, we constructed a mammalian expression plasmid encoding a codon-optimized 3×FLAG-TRP120; HEK293T cells were used because they have higher transfection efficiency than DH82 cells and could be readily infected with ΔTRP120 or WT *E. japonica* (Fig. 1F and G). ΔTRP120-infected HEK293T cells were transfected with the 3×FLAG-TRP120 plasmid at 1 d pi and harvested at 3 d pi. ΔTRP120 infection levels were similar between transfected cells expressing FLAG-TRP120 and sham-transfected control cells, as demonstrated by the morulae numbers/sizes in microscopic observations and band densities of *Ehrlichia* P28 by western blotting (Fig. 7A and B). Naïve mice were ip inoculated with TRP120- or sham-transfected HEK293T cells infected with ΔTRP120, and blood samples were collected daily from the submandibular venous plexus to assess the time course of bacteremia. In mice at 1 d pi and thereafter, ΔTRP120 from sham-transfected cells were almost undetectable in blood, whereas FLAG-TRP120 transfection significantly restored bacteremia up to 2 d pi (Fig. 7C). At 4 d pi, both groups of mice were moribund and, therefore, euthanized. Between the two groups, infection levels in the spleen and liver at 4 d pi did not differ significantly (Fig. 7D).

### ΔTRP120 *Ehrlichia japonica* cannot be effectively acquired by ticks from infected mice

**Fig 8:**
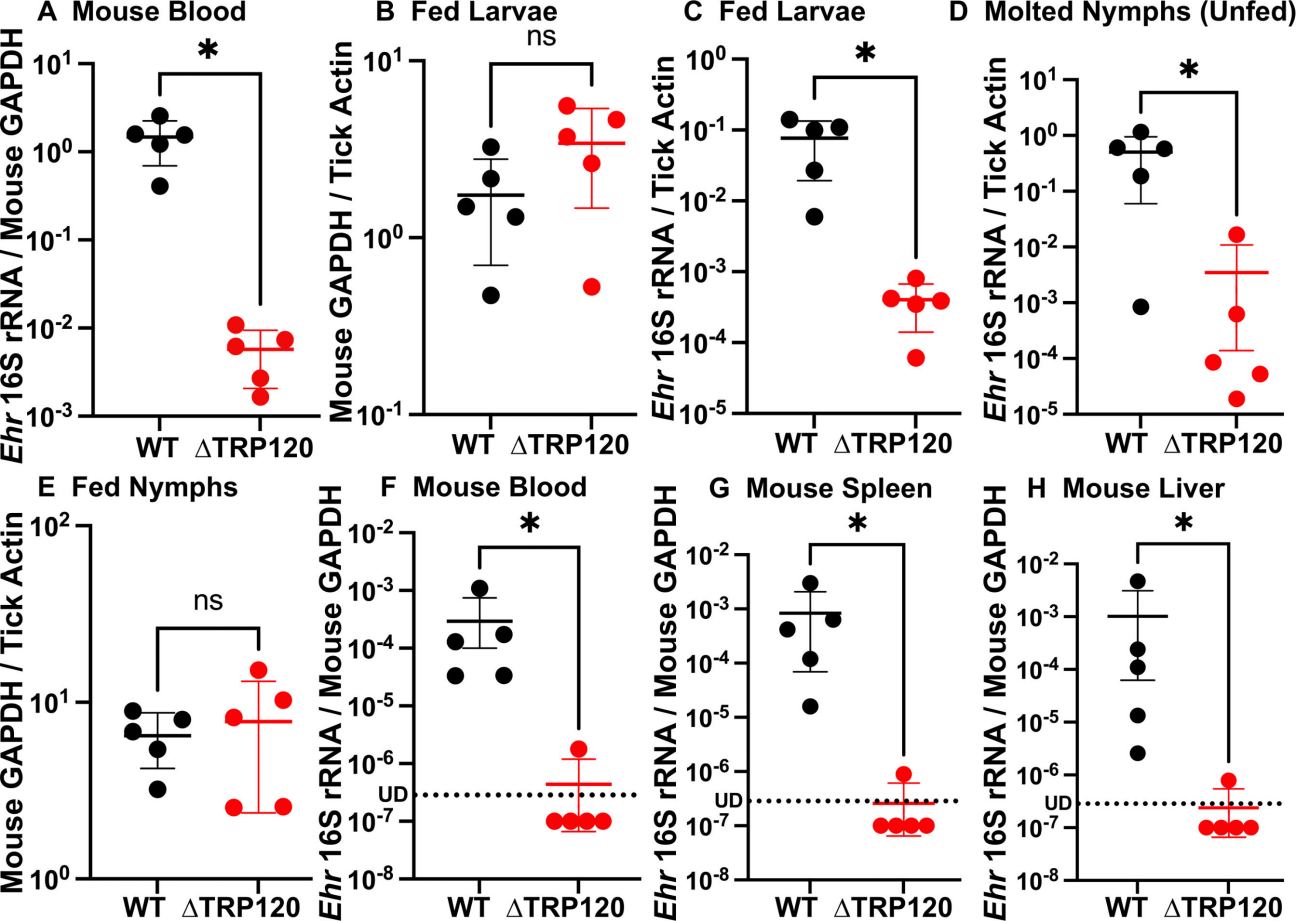
Mouse-to-tick and tick-to-mouse transmission of ΔTRP120 is significantly reduced compared with those of WT. (**A–D**) *I. scapularis* tick larvae were allowed to feed on mice infected with WT or ΔTRP120 *E. japonica* by ip inoculation at 3 d pi and removed at 7 d pi. *Ehrlichia* inoculum per mouse (WT, 2,385; ∆TRP120, 3,218) was determined by qPCR. (**A**) The amount of blood acquired by larvae was assessed by qPCR analysis using mouse GAPDH normalized by *I. scapularis* actin. (**B**) Blood samples were collected from WT or ΔTRP120-infected mice at 7 d pi, and the bacterial load was determined by qPCR analysis using *Ehrlichia* 16S rRNA normalized by mouse GAPDH. (**C, D**) DNA samples were extracted from fed larvae at 3 days after removal (**C**) or from nymphs molted from fed larvae for approximately 60 days after removal (**D**). The bacterial load was determined by qPCR analysis using *Ehrlichia* 16S rRNA normalized by *I. scapularis* actin. (**E–H**) Molted *I. scapularis* nymphs that acquired *E. japonica* infection as larvae were fed on naïve mice and removed from mice at day 4 post-feeding. (**E**) The amount of blood acquired by fed nymphs was determined by qPCR analysis using mouse GAPDH normalized by *I. scapularis* tick actin. (F–H) Bacterial load in the blood (**F**), spleen (**G**), and liver (**H**) of naïve mice fed upon by molted *I. scapularis* nymphs (infected as larvae) at day 7 post-feeding. qPCR analysis was performed using primers specific to *Ehrlichia* 16S rRNA and normalized against the level of mouse GAPDH. Data are presented as the mean ± SD (*N* = 5). ns, not significantly different; ^*^significantly different by Student’s *t*-test (*P* < 0.05). Red dots below the dotted line labeled with UD indicated undetectable by qPCR.

*E. japonica* is found in multiple *Ixodes* species of ticks (*I. ovatus, I. ricinus,* and *I. apronophorus*) (15, 18–20). As WT and ΔTRP120 could infect *I. scapularis* tick cells well and *I. ovatus* is not available in the US, we next developed a mouse model of tick-mediated (*I. scapularis*) transmission of *E. japonica*. Naïve mice were ip inoculated with ΔTRP120 or WT*,* and larval ticks were allowed to feed on them at 4 d pi and repleted at 7 d pi. The bacterial load of ΔTRP120 mutant in mouse blood at 7 d pi was significantly lower as forgoing studies without tick attachment (Fig. 8A). The ticks acquired the same amount of blood from mice infected with ΔTRP120 or WT, as demonstrated by the similar levels of mouse *GAPDH* in the ticks (Fig. 8B). Fed ticks were tested for bacterial infection immediately and after 3 days in the incubator to allow digestion of ingested blood. Tick acquisition of ΔTRP120 was significantly lower than that of WT (Fig. 8C). At 44–64 days post-repletion, fed larvae molted to nymphs, and the molting efficiency and timing were similar between ticks attached to mice infected with WT (131/209 ticks, 62.7%) or ΔTRP120 (130/190 ticks, 54.2%). Similar to fed larvae, the bacterial load of ΔTRP120 in molted nymphs was significantly lower than that of WT (Fig. 8D). Nonetheless, during transstadial transmission from fed larvae to molted nymphs, both WT and ∆TRP120 similarly replicated based on qPCR of *Ehrlichia* 16S rRNA per tick actin (WT, approximately sevenfold increase; ∆TRP120, approximately ninefold increase) (Fig. 8C and D).

Portions of molted *I. scapularis* nymphs were used to feed on naïve mice for 4 days, and these nymphal ticks infected with WT or ΔTRP120 acquired a similar amount of blood from mice (Fig. 8E). No weight loss or other clinical signs were observed in mice fed with WT or ΔTRP120-infected nymphs up to day 3 post-tick detachment (p.t.d.) (7 days post-tick attachment) when mice were euthanized to collect specimens. Bacterial loads of ΔTRP120 mutant in mouse blood, spleen, and liver were again significantly lower than those of WT (Fig. 8F through H). These results indicate that TRP120 is required for effective tick acquisition and subsequent transmission of *E. japonica*.

## DISCUSSION

The hallmark of human ehrlichiosis is white blood cell abnormalities that mimic the signs of leukemia, lymphoma, or endotoxemia, and consequently, cases are sometimes misdiagnosed as hematological malignancies, thrombotic thrombocytopenia purpura, or sepsis, causing delay in treatment (1, 9, 49–52). As shown in previous (16, 24, 25, 53) and current studies, *E. japonica* causes systemic infection and severe thrombocytopenia and increases serum alanine and aspartate aminotransferase abundance in infected mice, indicators that are similar to those observed for patients that progress to fatal HME (9). However, TRP120 is not required for infection or disease. *E. japonica* causes a toxic shock-like syndrome in mice by inducing the production of several Th1-type proinflammatory cytokines, such as TNF-α, IL-1β, IFN-γ, and IL-12p40 (22, 26–29, 54–56). Our results showed that TRP120 is not responsible for the induction of these cytokines. The role, if any, of the slight yet significantly more pronounced upregulation of IL-12p40 mRNA by ΔTRP120 mutant than WT in tissues remains unknown. Our results demonstrate that ehrlichiosis in mice is caused primarily by infection of tissues rather than blood, as ΔTRP120 was almost absent in the blood yet caused fatal ehrlichiosis that was indistinguishable from that caused by WT. This is in agreement with the previous report of M1-macrophage accumulation in the liver of *E. japonica*-infected mice (29).

Monocytes are heterogeneous circulating leukocytes that are poised to rapidly extravasate into inflamed tissues, such as those infected with *Ehrlichia*, to become macrophages (29, 57). When *Ehrlichia* infection induces more tissue inflammation, more blood monocytes are expected to extravasate into surrounding tissues. The striking disappearance of ΔTRP120 in the blood of mice starting ~16 h after iv inoculation suggests that TRP120 is required to retain infected monocytes in the blood by inhibiting extravasation. Alternative possibilities such as upon ΔTRP120 infection, only monocytes, but not macrophages, were perished or ΔTRP120 were killed only in monocytes, but not in macrophages, are unlikely, because of (i) early trends of greater infection of tissues with ΔTRP120 than WT, coinciding with the disappearance of ΔTRP120 from the blood (Fig. 5A); (ii) activation of TRP120 expression starting 12 h concurrently with the exponential growth of WT in blood vs disappearance of ΔTRP120 from blood vessels (Fig. 5A and B); and (iii) upregulation of monocyte extravasation-related surface markers on mouse peripheral blood monocytes incubated with the culture supernatant of ΔTRP120 (Fig. 6E).

Our results revealed that low levels of ΔTRP120 acquired by larvae led to a low level of molted nymph infection, which then led to low levels of transmission to naïve mice, indicating that the depletion of TRP120 in *E. japonica* can reduce transmission to a mammalian host by disrupting the natural lifecycle of *E. japonica*. In the current study, none of the mice infected by tick attachment showed clinical signs up to 3 days p.t.d. Also, WT numbers in the infected mice infected by tick attachment at day 7 were ~10,000-fold less than those infected by ip inoculation at day 7 (Fig. 4B and 8F). This is somewhat similar to the report by Saito and Walker that the mice fed with 10 nymphs infected with *E. muris-*like agent (EMLA, *E. muris* subspecies eauclairensis) had no signs of illness until day 7 p.t.d, and a few EMLA-infected tick-exposed mice (4 of 15 animals) died within 24 h after the onset of signs of illness between days 8 and 12 p.t.d. (58). It remains studied whether *E. japonica* causes a slow onset of clinical disease upon infected tick transmission like EMLA.

Although all tissues of mice inoculated with WT or ΔTRP120 that we examined were similarly infected with ΔTRP120 or WT, the *Ehrlichia* population in tissues, i.e., tissue macrophages, did not contribute to the tick-mediated acquisition of *Ehrlichia*. While several ehrlichial molecules are required for infection of mammalian or tick cells in culture (59, 60), or mammals (22, 61), ehrlichial molecules that drive the tick–mammal transmission are largely unknown. To our knowledge, TRP120 is the first *Ehrlichia* molecule shown to be required for tick acquisition of *Ehrlichia* species. Whether TRP120 is required for tick acquisition of *E. chaffeensis* remains unknown. However, a mutant of *E. chaffeensis* Arkansas with Himar1 insertion in TRP120 gene can infect ISE6 and DH82 cells, and *Amblyomma americanum* ticks by syringe inoculation as well as WT *E. chaffeensis* can (61, 62). Also, when dogs were inoculated (iv) with a pool of 17 *E. chaffeensis* mutants including the TRP120 mutant, this mutant was not found in canine blood (61). In conclusion, the development of a small-animal model of ehrlichiosis and tick transmission combined with a forward genetics approach will facilitate our understanding of the lifecycle of *Ehrlichia* species and the biomolecular mechanisms of ehrlichiosis and tick transmission.

## MATERIALS AND METHODS

### *Ehrlichia* and host cell culture

*E. japonica* (21) were cultured in DH82 cells in DMEM (Dulbecco minimal essential medium; Mediatech, Manassas, VA) supplemented with 5% fetal bovine serum (FBS) and 2 mM ʟ-glutamine with the addition of 0.1 µg/mL cycloheximide (Sigma, Burlington, MA). Uninfected and infected ISE6 cells were cultured in L15C300 medium as described (63, 64). Bacterial infection levels were determined by HEMA 3 staining (Thermo Fisher, Waltham, MA) and estimated under the microscope.

Monoclonal ΔTRP120 mutant was obtained by repeated limited dilution of H60-E2 mutant mixtures (22) in a 96-well flat-bottomed plate. Wells containing mCherry-expressing mutants were selected using a fluorescent microplate reader, and the cloned ΔTRP120 mutant was verified by PCR with primers flanking the insertion site of *TRP120* (EHF_0993) (22). The copy number of Himar1 insertion in the ΔTRP120 mutant was estimated by qPCR using primers for mCherry and *Ehrlichia* 16S rRNA genes normalized with pCis-mCherry-SS-Himar and *Ehrlichia* 16S rDNA pUC19 plasmids as standards.

### Cloning of rTRP120, antibodies, and immunofluorescence labeling

The gene encoding full-length *E. japonica TRP120* was codon-optimized for mammalian expression, custom-synthesized, and cloned into pCMV-3×FLAG-1A plasmid by GenScript (Piscataway, NJ). The codon-optimized *TRP120* was also cloned into pET33b(+) (Novagen, Gibbstown, NJ) to create a plasmid expressing 6×His-tagged TRP120 (rTRP120), and rTRP120 protein was affinity-purified from soluble fractions in transformed *E. coli* BL21(DE3) (New England Biolabs, Ipswich, MA) using HisPur Cobalt Resin (Thermo Fisher) as described (65). Mouse antiserum against TRP120 was developed in 10 C57BL/6 mice (Envigo, Indianapolis, IN), using rTRP120 protein bands separated by SDS-PAGE and homogenized with Quil A adjuvant (InvivoGen, San Diego, CA). The full list of antibodies used and the immunofluorescence labeling method are detailed in Supplemental Materials and Methods.

### Mouse inoculation and sample analysis

Female ICR mice were ip inoculated each with infected DH82 cells containing approximately 1,000–2,000 WT or ΔTRP120 *E. japonica*. Alternatively, male ICR mice were iv inoculated via retro-orbital plexus (66) with approximately 4–5 × 10^8^ host cell-free WT or ΔTRP120 *E. japonica*. Mice were monitored daily for clinical signs. At 4–7 d pi or other specific time points, mice were euthanized by CO_2_ inhalation followed by cervical dislocation, and organs including the liver, spleen, heart, and kidney were harvested. Blood samples were collected by cardiac puncture or submandibular venous plexus at specified time points. DNA and RNA were extracted from the blood and tissue samples and subjected to qPCR and RT-qPCR analysis. Complete blood count and mouse serum biochemistry profiles (liver function analysis) were performed at the OSU Comparative Pathology and Digital Imaging Shared Resource. For histopathology analysis, infected mouse tissues were fixed in 10% formalin, processed for paraffin embedding, sectioned at 4 µm, stained with hematoxylin and eosin or Wright Giemsa, and observed under light microscopy or a DeltaVision deconvolution microscope system.

### Transcomplementation of ΔTRP120

ΔTRP120 *E. japonica*-infected HEK293T cells were transfected with pCMV-3×FLAG-1A-TRP120 plasmid by electroporation in the Gene Pulser Xcell System (Bio-Rad). At 2 days post-transfection, infection was confirmed by HEMA 3 staining, and TRP120 expression was verified by western blotting. The infected cells containing ~10^6^ bacteria were ip inoculated into C57BL/6 mice (two groups, three mice per group). Blood samples were collected daily from the submandibular plexus from 1 to 3 d pi, and mice were euthanized on 4 d pi for terminal blood and tissue sample collection for qPCR analysis.

### Flow cytometry

Mouse PBMCs (4 × 10^5^ cells) were incubated with culture supernatants of ΔTRP120 or WT*-*infected or uninfected DH82 cells for 3, 6, and 9 h at 37°C. Harvested PBMCs were resuspended in 100 µL flow cytometry buffer and incubated with anti-mouse CD16/32 Fc blocker (BioLegend), then with APC anti-mouse CD11b (BioLegend) or FITC anti-mouse Ly6C antibodies (BioLegend). Stained cells were fixed and subjected to flow cytometry using the Attune NxT Flow Cytometer System (Thermo Fisher). Data were analyzed in FlowJo software (Ashland, OR).

### Tick attachment and transmission assay

Five 6-week-old ICR mice were each inoculated with 2,000–3,000 WT or ΔTRP120 from infected DH82 cells. At 3 d pi, approximately 50 *I. scapularis* larval ticks (Oklahoma State University Tick Rearing Facility; Stillwater, OK) were placed inside a tick confinement chamber glued on the shaved back of each mouse under inhalational anesthesia. At day 3 after tick attachment (7 d pi), 30–50 engorged larval ticks were recovered from each mouse, and all mice were euthanized for blood and tissue sample collection. Three engorged larval ticks from each mouse were pooled and processed for DNA extraction and qPCR. The remaining ticks were incubated at 21°C until they molted (~2 months). One molted nymphal tick from each mouse was processed for DNA extraction and qPCR, and five to eight molted nymphal ticks were attached to each naïve female 6-week-old ICR mouse. Engorged ticks were removed on day 4 after tick attachment, and all mice were euthanized on day 7 after tick attachment to collect blood and tissue samples.

Additional experimental details, including culture, cloning, mouse sample analysis, extracellular TRP120 detection, RT-qPCR, and statistical analysis, are described in Supplemental Materials and Methods.

## SUPPLEMENTAL MATERIAL

Supplemental material mbio.00476-24-s0001.docx Supplemental materials and methods; Table S1.

ASM does not own the copyrights to Supplemental Material that may be linked to, or accessed through, an article. The authors have granted ASM a non-exclusive, world-wide license to publish the Supplemental Material files. Please contact the corresponding author directly for reuse.
