# Supplementary material for: Ehrlichia effector TRP120 manipulates bacteremia to facilitate tick acquisition
Source: mBio. 2024 Mar 19;15(4):e00476-24. doi: 10.1128/mbio.00476-24 (PMC11005420; doi:10.1128/mbio.00476-24)
Supplement: Supplemental material — s and methods; Table S1. [file mbio.00476-24-s0001.docx]

# Supplemental Materials and Methods

**Ethics statement.** All animal experiments were performed in accordance with guidelines of the Ohio State University Institutional Animal Care and Use Committee, which also approved the experimental protocol. The university program has full accreditation by the Association for Assessment and Accreditation of Laboratory Animal Care International under 000028 and has Public Health Services assurance renewal A3261-01. The program is licensed by the USDA, number 31-R-014, and is in full compliance with Animal Welfare Regulations.

**Culture of host cells and their infection with *Ehrlichia*.** Uninfected DH82 cells were cultured in DMEM (Dulbecco minimal essential medium; Mediatech, Manassas, VA) supplemented with 5% fetal bovine serum (FBS; Atlanta Biologicals, Lawrenceville, GA) and 2 mM ʟ-glutamine (ʟ-Gln; Gibco, Waltham, MA). DH82 cells infected with wild-type (WT) *E. japonica* strain HF (1) or ΔTRP120 mutant (H60-E2) (2) were cultured with the addition of 0.1 μg/ml cycloheximide (Millipore Sigma, Burlington, MA). Uninfected and infected HEK293T cells (ATCC, Manassas, VA) were cultured in DMEM supplemented with 8% FBS and 2 mM ʟ-Gln. Both DH82 and HEK293T cells were cultured at 37°C under 5% CO_2_ in a humidified atmosphere. Uninfected and infected ISE6 cells were cultured in L15C300 medium as described (3, 4). Bacterial infection levels were determined by HEMA3 staining (Thermo Fisher, Waltham, MA) and estimated under the microscope.

## Cloning ΔTRP120 mutant and clonality confirmation. DH82 cells were seeded at 10^4^ cells in 50 μl DMEM medium per well in a 96-well flat-bottomed plate (Greiner, Monroe, NC) and inoculated with approximately three H60-E2-infected DH82 cells in each well. After overnight incubation, 150 μl of additional culture medium was added to each well and cells were allowed to grow to confluency for ~5 days. Cells were scraped off using sterile pipette tips and ~10^4^ cells in 20 μl were transferred to a new 96-well flat, clear-bottom black plate (Tecan, Morrisville, NC) until cells reached confluency. mCherry-expressing mutant was detected using a Tecan Infinite M Nano+ microplate reader (Tecan) at the excitation wavelength of 560 nm and emission wavelength of 635 nm, and a fluorescence reading of three standard deviations higher than the control uninfected DH82 cells was considered positive. Positive wells were expanded and checked for clonality by PCR using primers flanking the target insertion site at genomic locus of 1,142,873 bp (Fig. 1) (2). The cloned ΔTRP120 or WT–infected DH82 cells were examined for TRP120 protein expression by western blot analysis using mouse antiserum against rTRP120, and the copy number of Himar1 insertions in the ΔTRP120 mutant was estimated by qPCR was performed using primers for mCherry and *Ehrlichia* 16S rRNA gene, and pCis-mCherry-SS Himar A7 and *Ehrlichia* 16S rDNA pUC19 plasmids as standards. All primers used in this study are listed in Table S1.

## Cloning of rTRP120. The gene encoding full-length *E. japonica* *TRP120* (EHF_0993) was codon-optimized for expression in mammalian cells, custom-synthesized, and cloned into pCMV-3×FLAG-1A plasmid by GenScript (Piscataway, NJ). The codon-optimized *TRP120* was PCR amplified and cloned into pET33b(+) (Novagen, Gibbstown, NJ) at the NcoI and XhoI restriction sites to create a plasmid expressing 6×His-tagged TRP120 (rTRP120). rTRP120 protein was affinity purified from soluble fractions in transformed *E. coli* BL21(DE3) (New England Biolabs, Ipswich, MA) using HisPur Cobalt Resin (Thermo Fisher) as described (5).

## Antibodies and immunofluorescence staining. Mouse antiserum against TRP120 was developed in ten C57BL/6 male mice (Envigo, Indianapolis, IN), using rTRP120 protein bands separated by SDS-PAGE and homogenized with Quil A adjuvant (InvivoGen, San Diego, CA). Mice were immunized intraperitoneally three times at two-week intervals, and mouse serum was collected 10 days after the last immunization. Other antibodies used in this study include rabbit anti-P28 (6), rabbit anti-β-actin (Sigma-Aldrich, St. Louis, MO), Alexa Fluor (AF) 488–conjugated rat monoclonal anti-FLAG (BioLegend, San Diego, CA), AF488-conjugated goat anti-mouse IgG and anti-rabbit IgG (Invitrogen, Carlsbad, CA). The cell-permeant Hoechst 33342 (Invitrogen) was used to stain nucleic acid. Fluorescence images with overlay differential interference contrast images (DIC) were acquired and analyzed with a DeltaVision PersonalDV deconvolution microscope system (GE Healthcare Life Sciences, Marlborough, MA).

## Mice inoculation and sample collection. Female ICR mice at 6 weeks old (2 groups with 5 mice each) were ip inoculated each with infected DH82 cells containing approximately 1,000 – 2,000 WT or ΔTRP120 resuspended in 0.5 ml serum-free DMEM using a 1-ml Tuberculin syringe with a 26-gauge needle (BD, Franklin Lakes, NJ). Alternatively, male ICR mice were iv inoculated via retro-orbital plexus (7), each with approximately 4–5 × 10^8^ host cell–free WT or ΔTRP120 in 150 µl serum-free DMEM using a 0.5-ml insulin syringe with a 28-gauge micro-fine needle (BD). Mice were monitored daily for clinical signs. Blood samples were collected in Covidien Monoject with 7.5% Solution EDTA (K3) 0.04 ml Liquid Additive tubes (Thermo Fisher) by cardiac puncture or submandibular venous plexus at specified time points. At 4–7 dpi or other specific time points, mice were euthanized by CO_2_ inhalation followed by cervical dislocation and organs including liver, spleen, heart, and kidney were harvested. Half of the collected blood and tissue samples were stored at –20°C for DNA extraction, and the other half of the samples were preserved in RNAlater buffer (Qiagen, Germantown, MD) and stored at –80°C until RNA extraction.

## Complete blood count and liver function analysis, and histopathology. ICR mice at 6 weeks old (3 groups, 4 mice per group) were each IP inoculated with WT or ΔTRP120–infected (~1,000 bacteria/mouse) or uninfected DH82 cells. At 7 dpi, blood samples were collected by cardiac puncture under inhalational anesthesia with 2% (v/v) isoflurane (Akorn Pharmaceuticals, Lake Forest, IL), with 200 µl in Dipotassium Microgard™ Closure Dipotassium EDTA Tube (BD Biosciences, San Jose, CA) for automated complete blood count and 800 µl in Microgard™ Closure Serum Separator Tube (BD Biosciences) for mouse serum biochemistry profiles (mouse liver function panel) at Ohio State University Comparative Pathology and Digital Imaging Shared Resource. Infected mouse tissues were fixed in 10% neutral buffered formalin, processed for paraffin embedding, sectioned at 4 µm, stained with hematoxylin and eosin or Wright Giemsa, and observed under light microscopy or a DeltaVision deconvolution microscope system. Total 6 mm^2^ area (6 each image of 1 mm^2^, 10× objective lens) each from WT and ΔTRP120–infected liver sections were evaluated and distinct area of lesions showing necrosis of multiple hepatocytes (characterized by pyknotic nuclei, hyper-eosinophilic cytoplasm, and/or loss of cellular integrity) with or without inflammatory infiltrates were scored.

**qPCR and RT-qPCR.** DNA was extracted from samples using DNeasy Blood & Tissue kits (Qiagen), and RNA was isolated using RNeasy Mini kits (Qiagen). cDNA was synthesized from 1–2 µg of extracted RNA using the Maxima H minus First Strand cDNA synthesis kit (Thermo Fisher) with random hexamer primers. qPCR and RT-qPCR analysis were performed using Maxima SYBR Green/ROX Master Mix (Thermo Fisher) according to the manufacturers' protocols in Mx3000P (Stratagene, Waltham, MA) or AriaMx Real-time PCR system (Agilent, Santa Clara, CA). To quantify the exact inoculums of *E. japonica*, an absolute quantification method was used by creating a standard curve of *Ehrlichia* 16S rDNA cloned into plasmid pUC19 as a standard. Infectivity of WT or ∆TRP120 in DH82, ISE6, and HEK293T cells were quantified by RT-qPCR by inoculating the host cells at multiplicity of infection (MOI) of 100-200 and harvesting at 2-3 d pi. To quantify relative *E. japonica* loads in mouse blood and tissues, or ticks, qPCR was performed using primers specific for *Ehrlichia* 16S rDNA and mouse *GAPDH* or *I. scapularis* *Actin* (8). To assess cytokine gene expression, RT-qPCR was performed using primers specific for mouse cytokine mRNAs along with mouse *GAPDH* for normalization.

## Trans-complementation of ΔTRP120*.* HEK293T cells were infected with ΔTRP120. At log phase of *E. japonica* growth (~30% infected cells), HEK293T cells were resuspended in 100 µl Opti-MEM Medium (Gibco) at 5 × 10^6^ cells/ml, and transfected with 15 µg endo-free pCMV-3×FLAG-1A-TRP120 plasmid (GenScript, Piscataway, NJ) by using electroporation at 100 V, 1000 μF, ∞ Ω in Gene Pulser Xcell System (Bio-Rad). ΔTRP120–infected HEK293T cells and control sham-transfected infected cells were harvested at 2 days post-transfection. Infection was confirmed by HEMA 3 staining, and TRP120 expression was verified by western blot analysis. The infected cells containing ~10^6^ bacteria were IP inoculated into C57BL/6 mice (2 groups, 3 mice per group). Blood samples were collected daily from the submandibular plexus from 1 to 3 dpi, and mice were euthanized on 4 dpi for terminal blood and tissue sample collection for qPCR analysis.

## Analysis of extracellular TRP120. Protein concentrations of purified rTRP120 were determined by western blot analysis with bovine serum albumin as standard (Thermo Fisher) and serially diluted rTRP120 standards from 0.125 to 2 ng/µl. The culture supernatant was harvested from uninfected and *E. japonica*–infected DH82 cells following centrifugation at 15,000 × *g* for 10 min at 4°C to remove cellular debris or released bacteria. Infected DH82 cells were lysed in RIPA Buffer (Thermo Scientific). Samples along with diluted rTRP120 standards were subjected to 7.5% SDS-PAGE and Western blot analysis using mouse sera against rTRP120 and peroxidase-conjugated secondary antibody (SeraCare Life Sciences; Milford, MA). Reacting bands were visualized with Pierce ECL Western Blotting Substrate (Thermo Fisher) and images were captured using Amersham AI680QC gel documentation system (GE Healthcare). Protein band densities were quantified by ImageJ (National Institutes of Health, Bethesda, MD).

**Flow cytometry.** Blood samples were collected in Covidien Monoject with 7.5% Solution EDTA (K3) 0.04-mL Liquid Additive tubes (Thermo Fisher), and proceeded for PBMC isolation by using Ficoll-Paque PLUS Density Gradient Media (Cytiva, Marlborough, MA) and 4 ×10^5^ cells in 100 µl of 10% FBS and 2 mM ʟ-Gln were incubated with 100 µl of the culture supernatants of ~90% ΔTRP120 or WT*-*infected, or uninfected DH82 cells in a Costar 24-well plate for 3, 6, and 9 h at 37°C. Harvested PBMC were resuspended in 100 µl flow cytometry buffer (PBS with 2% FBS, 1% BSA, and 0.1% NaN_2_). Cell suspensions were incubated with TruStain FcX™ PLUS anti-mouse CD16/32 Fc blocker antibody (BioLegend) for 10 mins at 4 °C with end-to-end rotation, then incubated with APC anti-mouse/human CD11b antibody (BioLegend) or FITC anti-mouse Ly6C antibody (BioLegend) for 1 h at 4 °C with end-to-end rotation. After washing three times with 500 µl flow cytometry buffer, stained cells were fixed in 4% paraformaldehyde and incubated for 30 mins at 4°C, and subjected for flow cytometry using the Attune™ NXT Flow Cytometer System (Thermo Fisher). Data were analyzed in FlowJo software (Ashland, OR).

**Tick attachment.** Five female ICR mice at 6 weeks old (Envigo) were each inoculated with 2,000 – 3,000 WT or ΔTRP120 from infected DH82 cells. The back of each mouse was shaved and cleaned. Two plastic rings (diameter 5 mm, height 2 mm) were cut from a 1.5-microcentrifuge tube (AE Bios, Cincinnati, OH), and clasped together with a nylon mesh (2 cm × 2 cm; ELKO Filtering Co, Switzerland) to make a tick confinement chamber. Approximately 50 *I. scapularis* larval ticks (Oklahoma State University Tick Rearing Facility; Stillwater, OK) were placed inside of the tick confinement chamber which was glued using Animal ID Tag Cement (NASCO, Detroit, MI) on the shaved back of each mouse under inhalational anesthesia at 4 d pi. At day 3 after tick attachment (7 d pi), 30–50 engorged larval ticks were recovered from each mouse, and all mice were euthanized for blood and tissue sample collection as described above. Three engorged larval ticks from each mouse were pooled, homogenized by using tissue grinders attached on a pellet pestle cordless motor (Sigma-Aldrich) and processed for DNA extraction and qPCR. Remaining ticks were incubated at 21°C and >70% relative humidity with a 10 h dark/14 h light cycle until they molted (~2 months). One molted nymphal tick from each mouse (5 per group, 2 groups) was processed for DNA extraction and qPCR. Five to eight molted nymphal ticks from each mouse were attached to each of naïve female ICR mice at 6 weeks old (Envigo) (5 per group, 2 groups). Engorged ticks were removed at day 4 after tick attachment, and all mice were euthanized at day 7 after tick attachment to collect blood and tissue samples.

**Statistical analysis.** The significance of differences between values was assessed using the two-tailed Student *t* test or analysis of variance. Data analysis and figure drawing were performed with Prism 9 software (GraphPad, San Diego, CA).

# Supplemental Table S1. Primers used in this study. *

| **Target** | **Primer sequence (5’-3’)** | **Note** |
| --- | --- | --- |
| **TRP120 cloning ^†^** | | |
| Codon-optimized *TRP120* * | F: GAT CCATGG ATATCGACAACAACATC  R: TCA CTCGAG GATGATGCTATTCACGGCGTTG | For cloning codon-optimized *TRP120* into *Ncol* (F) and *Xhol* (R) sites on pET33b (+) plasmid. |
| **Flank PCR** | | |
| *E. japonica*  i-1142873 * | F: CAAAATTGCACGCACAAATATGC  R: CAATCTACTGGACATACCTCAACAC | For flanking the Himar1 insertion site i-1142873 of *E. japonica* ΔTRP120. |
| ***Ehrlichia* quantification** | | |
| *Ehr* 16S rRNA (9) | F: CGGGGGAAAGATTTATCGCTATTA  R: CGCTTGCCCCCTCCGTATTA | For qPCR and RT-qPCR to quantify *Ehrlichia.* |
| Mouse GAPDH (10) | F: GTTGTCTCCTGCGACTTCA  R: GGTGGTCCAGGGTTTCTTA | For qPCR and RT-qPCR to normalize quantification in mouse. |
| *I. scapularis* actin (11) | F: GCCCTGGACTCCGAGCAG  R: CCGTCGGGAAGCTCGTAGG | For qPCR to normalize quantification in *I. scapularis*. |
| *I. scapularis* actin (8, 12) | F: CTTGGAGGGAGACATCTTTGTG  R: CGCTCCATGTCATTCCAATCT | For RT-qPCR to normalize quantification in ISE6. |
| Canine GAPDH (12) | F: ATCACTGCCACCCAGAAGAC  R: TCAGCTCAGGGATGACCTTG | For qPCR and RT-qPCR to normalize quantification in DH82. |
| Human Actin (13) | F: AGAGCTACGAGCTGCCTGAC  R: AGCACTGTGTTGGCGTACAG | For RT-qPCR to normalize quantification in HEK293T. |
| Himar1 mCherry * | F: CTCCGGGTACATTCTCTCGC  R: GGAACGCGTCATGAACTTCG | For qPCR to quantify copy of Himar1 insertion in *E. japonica* ΔTRP120 |
| **Mouse cytokine mRNA expression ^5^** | | |
| Mouse  TNF-α (14) | F: CATCTTCTCAAAATTCGAGTGACAA  R: TGGGAGTAGACAAGGTACAACCC | For RT-qPCR to check TNF-α mRNA expression in mouse. |
| Mouse  IFN-γ (14) | F: GCGTCATTGAATCACACCTG  R: TGAGCTCATTGAATGCTTGG | For RT-qPCR to check IFN-γ mRNA expression in mouse. |
| Mouse  IL-1β (14) | F: GGGCCTCAAAGGAAAGAATC  R: TACCAGTTGGGGAACTCTGC | For RT-qPCR to check IL-1β mRNA expression in mouse. |
| Mouse  IL-10 (14) | F: GGTTGCCAAGCCTTATCGGA  R: ACCTGCTCCACTGCCTTGCT | For RT-qPCR to check IL-10 mRNA expression in mouse. |
| Mouse  IL-12 *p40* (14) | F: AAGGAACAGTGGGTGTCCAG  R: CATCTTCTTCAGGCGTGTCA | For RT-qPCR to check IL-12 mRNA expression in mouse. |

* Primers were designed in this study.

^†^ F, forward; R, reverse complement primers; underlined sequences, restriction enzyme sites.

**Reference:**

1. Lin M, Xiong Q, Chung M, Daugherty SC, Nagaraj S, Sengamalay N, Ott S, Godinez A, Tallon LJ, Sadzewicz L, Fraser C, Dunning Hotopp JC, Rikihisa Y. 2021. Comparative Analysis of Genome of Ehrlichia sp. HF, a Model Bacterium to Study Fatal Human Ehrlichiosis. BMC Genomics 22:11.

2. Bekebrede H, Lin M, Teymournejad O, Rikihisa Y. 2020. Discovery of in vivo Virulence Genes of Obligatory Intracellular Bacteria by Random Mutagenesis. Front Cell Infect Microbiol 10:2.

3. Munderloh UG, Liu Y, Wang M, Chen C, Kurtti TJ. 1994. Establishment, maintenance and description of cell lines from the tick Ixodes scapularis. J Parasitol 80:533-43.

4. Munderloh UG, Jauron SD, Fingerle V, Leitritz L, Hayes SF, Hautman JM, Nelson CM, Huberty BW, Kurtti TJ, Ahlstrand GG, Greig B, Mellencamp MA, Goodman JL. 1999. Invasion and intracellular development of the human granulocytic ehrlichiosis agent in tick cell culture. J Clin Microbiol 37:2518-24.

5. Kumagai Y, Cheng Z, Lin M, Rikihisa Y. 2006. Biochemical activities of three pairs of *Ehrlichia chaffeensis* two-component regulatory system proteins involved in inhibition of lysosomal fusion. Infect Immun 74:5014-22.

6. Ohashi N, Zhi N, Zhang Y, Rikihisa Y. 1998. Immunodominant major outer membrane proteins of *Ehrlichia chaffeensis* are encoded by a polymorphic multigene family. Infect Immun 66:132-139.

7. Yardeni T, Eckhaus M, Morris HD, Huizing M, Hoogstraten-Miller S. 2011. Retro-orbital injections in mice. Lab Anim (NY) 40:155-60.

8. Budachetri K, Teymournejad O, Lin M, Yan Q, Mestres-Villanueva M, Brock GN, Rikihisa Y. 2020. An Entry-Triggering Protein of Ehrlichia Is a New Vaccine Candidate against Tick-Borne Human Monocytic Ehrlichiosis. mBio 11.

9. Niu H, Rikihisa Y, Yamaguchi M, Ohashi N. 2006. Differential expression of VirB9 and VirB6 during the life cycle of *Anaplasma phagocytophilum* in human leucocytes is associated with differential binding and avoidance of lysosome pathway. Cell Microbiol 8:523-34.

10. Prima V, Kaliberova LN, Kaliberov S, Curiel DT, Kusmartsev S. 2017. COX2/mPGES1/PGE(2) pathway regulates PD-L1 expression in tumor-associated macrophages and myeloid-derived suppressor cells. Proceedings of the National Academy of Sciences of the United States of America 114:1117-1122.

11. Hojgaard A, Lukacik G, Piesman J. 2014. Detection of Borrelia burgdorferi, Anaplasma phagocytophilum and Babesia microti, with two different multiplex PCR assays. Ticks Tick Borne Dis 5:349-51.

12. Tamura Y, Ohta H, Yokoyama N, Lim SY, Osuga T, Morishita K, Nakamura K, Yamasaki M, Takiguchi M. 2014. Evaluation of selected cytokine gene expression in colonic mucosa from dogs with idiopathic lymphocytic-plasmacytic colitis. J Vet Med Sci 76:1407-10.

13. Song Y, Zuo Y. 2014. Occurrence of HHIP gene CpG island methylation in gastric cancer. Oncol Lett 8:2340-2344.

14. Miura K, Rikihisa Y. 2009. Liver transcriptome profiles associated with strain-specific *Ehrlichia chaffeensis*-induced hepatitis in SCID mice. Infect Immun 77:245-54.
